# Foot pain and inflammatory markers: a cross sectional study in older adults

**DOI:** 10.1186/s13047-022-00565-0

**Published:** 2022-08-08

**Authors:** Anna C. Siefkas, Alyssa B. Dufour, Yvonne M. Golightly, Hylton B. Menz, Howard J. Hillstrom, Marian T. Hannan

**Affiliations:** 1 Department of Epidemiology, Harvard T.H. Chan School of Public Health, Boston, MA 02115 USA; 2 Hinda and Arthur Marcus Institute for Aging Research, Hebrew SeniorLife, Boston, MA USA; 3 Department of Medicine, Beth Israel Deaconess Medical Center, Harvard Medical School, Boston, MA USA; 4 Department of Epidemiology, University of North Carolina, Chapel Hill, NC USA; 5 Thurston Arthritis Research Center, University of North Carolina, Chapel Hill, NC USA; 6 School of Allied Health, Human Services and Sport, La Trobe University, Bundoora, VIC Australia; 7 Hospital for Special Surgery, New York, NY USA

**Keywords:** Foot pain, Foot disorders, Inflammation, Epidemiology, Aging, Cohort study

## Abstract

**Background:**

Foot disorders may limit independence and reduce quality of life for older adults. Obesity is a risk factor for foot conditions; both mechanical load and metabolic effects may contribute to these conditions. This study determined cross-sectional associations between inflammatory markers and foot disorders.

**Methods:**

Participants were drawn from the Framingham Foot Study (2002–2008). C-reactive protein (CRP), interleukin-6 (IL-6), and tumor necrosis factor alpha (TNF-α) were each examined for associations with foot pain, forefoot pain, hindfoot pain, hallux valgus, hallux rigidus, and toe deformities (claw, hammer, or overlapping toes). Unadjusted and adjusted (age, body mass index, physical activity, smoking status) sex-specific logistic regression was performed.

**Results:**

Of 909 participants, 54% were women (mean age 65 $\pm$ 9 years), 20% had foot pain, 29% had hallux valgus, 3% had hallux rigidus, and 27% had toe deformities. In unadjusted models, higher CRP (OR [95% CI] = 1.5 [1.1, 2.0]) and IL-6 (OR [95% CI] = 1.8 [1.2, 2.6]) were associated with foot pain among men; higher CRP was associated with foot pain (OR [95% CI] = 1.3 [1.0, 1.5]) among women. Higher CRP (OR [95% CI] = 1.9 [1.1, 3.2]) and IL-6 (OR [95% CI] = 2.4 [1.2, 4.7]) were associated with forefoot pain in men. Higher CRP was associated with hindfoot pain ([95% CI] = 1.8 [1.2, 2.6]) in women. After adjustment, CRP ([95% CI] = 1.5 [1.1, 2.0]) and IL-6 ([95% CI] = 1.8 [1.2, 2.6]) remained associated with foot pain in men, and IL-6 with forefoot pain ([95% CI] = 2.9 [1.4, 6.1]) in men. No associations with structural foot disorders were observed.

**Conclusions:**

Inflammation may impact foot pain. Future work assessing whether inflammation is part of the mechanism linking obesity to foot pain may identify areas for intervention and prevention.

**Supplementary Information:**

The online version contains supplementary material available at 10.1186/s13047-022-00565-0.

## Introduction

Foot pain and structural foot disorders can impair mobility, particularly in older adults. Studies have found that that foot pain is associated with self-reported [1] and objectively assessed [2] reductions in mobility and weight bearing tasks, as well as reduced ability to perform instrumental activities of daily living (IADL) [3]. As a result, problems with foot pain and posture increase disability, reduce quality of life, and result in loss of independence [1–3].

Increased body mass index (BMI) and fat mass are associated with various presentations of foot pain [4, 5], which some studies have attributed to increased mechanical load [6]. However, this pathway is unable to explain certain findings, such as the fact that fat mass and fat mass index have been associated with foot pain independently of fat free mass, while other measures of body mass have not been associated with foot pain after adjustment for fat mass [4, 7, 8]. Increased BMI is also associated with increased risk of related conditions like osteoarthritis (OA) of the hand, an association which cannot be attributed to mechanical load alone [9–11]. Understanding the full likely range of mechanisms linking obesity and foot pain may enable more targeted treatment and prevention strategies.

The metabolic effects of obesity may also explain some relationships between obesity and musculoskeletal pain. A large body of literature has emerged describing the metabolic changes associated with obesity [12]. Obesity has been linked to increased production of several cytokines, including C-reactive protein (CRP), tumor necrosis factor alpha (TNF-α), and interleukin-6 (IL-6) [13]. Previous research has linked inflammation generally and cytokines to pain, and to conditions like OA which involve both pain and structural alterations [14, 15]. However, little research has been conducted exploring the relationship of inflammatory markers with foot pain and foot disorders, and the presence or absence of a relationship has yet to be clearly established. In the North West Adelaide Health Study, IL-6 and TNF-α were assessed for their associations with prevalent and incident foot pain. Levels of both IL-6 and TNF-α were higher among those with prevalent foot pain or future foot pain when compared to those without foot pain, and the association between IL-6 and prevalent foot pain approached significance (*p* = 0.057) [16].

With the aim of furthering understanding about potential metabolic links between obesity and foot pain, this cross-sectional study aims to assess whether CRP, TNF-α, and IL-6 are associated with prevalent foot pain and structural foot disorders.

## Methods

### Study participants

This study included participants from the Framingham Foot Study (FFS), comprised of members of the Framingham Heart Study (Offspring Cohort) who completed the FFS examination during 2002–2008. The FFS was approved by the Institutional Review Boards (IRBs) at Boston University and Hebrew SeniorLife, and all participants provided written informed consent prior to enrollment. Participants in the FFS received a physical examination of the foot to assess structural disorders and were queried on presence and locations of foot pain. Participants provided information on health status, history, and symptoms through a structured questionnaire. Each participant received a single FFS examination and contributed one observation to the present analysis.

We included only FFS participants with available data on CRP, IL-6, BMI, age, sex, and physical activity index (PAI). Because many participants were missing TNF-α, participants were included with or without TNF-α (Supplementary Fig. 1).

### Foot pain and structural foot disorders

Foot pain was assessed with the question “On most days do you have pain, aching, or stiffness in either of your feet?” Answers were dichotomized as yes (pain in one or both feet) and no (no pain in either foot). Forefoot and hindfoot pain were identified by asking participants to locate areas of pain on a picture of the foot.

Feet were examined to determine the presence of hallux valgus, hallux rigidus, hammer toes, claw toes, or overlapping toes [5]. For this analysis, toe deformities were considered present if a hammer, claw, or overlapping toes were present on one or both feet. Hallux valgus and hallux rigidus were each considered present if present on one or both feet.

### Inflammatory markers

Information on CRP, TNF-α, and IL-6 was obtained from Framingham Offspring Study visits from 1998—2001. Although these visits preceded FFS visits by 1 to 7 years, prior analyses of longitudinal CRP measurements in the Framingham Offspring Cohort have found measurements to be fairly stable over short and long-term periods averaging 4 and 16 years, respectively [17]. Similar stability has been noted for IL-6 and TNF-α in other populations [18].

Inflammatory markers were measured from fasting blood draws. CRP was measured through particle enhanced immunonephelometry, TNF-α through enzyme immunoassay, and IL-6 through a quantitative enzyme-linked immunosorbent assay. Because the distributions of all inflammatory markers were right skewed, values were log-transformed before modeling.

A normal CRP range has been proposed by the American Board of Internal Medicine as < 8 mg/L [19], however, internal reference ranges vary by institution. Normal ranges for IL-6 and TNF-$\alpha$ have yet to be established. Higher values for all three markers indicate a greater degree of systemic inflammation.

### Covariates

Age (in years), current smoking status (yes or no), BMI, and physical activity index (PAI) were measured as potential confounders. The PAI is a validated weighted score of usual metabolic activity in a 24-h period, and ranges from 24 to 120 (lowest to highest activity). Covariates were obtained at the time of the FFS exam.

### Statistical analysis

Distributions of inflammatory markers, foot outcomes, and confounders were calculated for the whole population and separately for male and female participants. Prior literature has found that incidence of and risk factors for foot pain vary by sex [20]. Therefore sex-specific logistic regression models were used to evaluate unadjusted and adjusted (age, smoking status, BMI, and PAI) relationships between each inflammatory marker and each foot outcome, independently. Covariates were selected for inclusion based on prior literature and *a priori* hypotheses about confounding relationships. These covariates were included in the model regardless of their statistical significance with the outcome. Because some participants were missing data on TNF-α level, TNF-α models were evaluated on a subset of the full study population.

### Sensitivity analysis

For each pain outcome (foot, forefoot, or hindfoot), we tested interactions between BMI and each inflammatory marker to determine whether associations varied by BMI.

## Results

In total, 909 FFS participants were included in this analysis, 46% of whom were men (Table 1). TNF-α data were available for 647 (71%) participants (46% men). Foot pain, forefoot pain, and hallux valgus were more prevalent in women, while hallux rigidus was more common in men. Additionally, 163 participants reported knee OA symptoms (93 women and 70 men).

**Table 1:** Study population characteristics. Mean $\pm$ S.D. or N (%) given

	All **(*n* = 909)**	**Men** **(*n* = 421)**	Women **(*n* = 488)**
**Age (years)** **range: 36–89**	65.3 $\pm$ 9.2	65.6 $\pm$ 9.1	65.0 $\pm$ 9.3
**Current smoker**	76 (8.4%)	37 (8.8%)	39 (8.0%)
**BMI (kg/m**^**2**^**)**	28.7 $\pm$ 5.4	29.1 $\pm$ 4.56	28.2 $\pm$ 6.0
**Physical Activity Index** **range: 26.5–70.8**	37.8 $\pm$ 6.03	38.4 $\pm$ 6.5	37.3 $\pm$ 5.5
**Knee pain**	163 (17.9%)	70 (16.6%)	93 (19.1%)
**CRP**^**ad**^** (mg/L)**	2.00 (0.93 – 4.60)	1.65 (0.85 – 3.53)	2.41 (1.05 – 5.75)
**IL-6**^**ad**^** (pg/mL)**	2.47 (1.75 – 3.93)	2.58 (1.73 – 3.93)	2.39 (1.76 – 3.87)
**TNF-α**^**abd**^** (pg/mL)**	1.15 (0.87 – 1.53)	1.16 (0.89 – 1.49)	1.15 (0.84 – 1.56)
**Foot pain**	181 (19.9%)	63 (15.0%)	118 (24.2%)
**Forefoot pain**	67 (7.4%)	12 (2.9%)	55 (11.3%)
**Hindfoot pain**	51 (5.6%)	25 (5.9%)	26 (5.3%)
**Hallux valgus**	260 (28.6%)	89 (21.1%)	171 (35.0%)
**Hallux rigidus**	26 (2.9%)	17 (4.0%)	9 (1.8%)
**Any toe deformity**^**c**^	244 (26.8%)	114 (27.1%)	130 (26.6%)

*Abbreviations: BMI* Body mass index, *CRP* C-reactive protein, *IL-6* Interleukin-6, *TNF-α* Tumor necrosis factor alpha

^a^ Median (IQR)

^b^ *N* = 647; 299 men, 348 women

^c^ Toe deformity includes claw toe, hammer toe, or overlapping toes on either foot

^d^ Normal range of inflammatory markers is as follows. CRP: < 8 mg/L. IL-6: not established. TNF-$\alpha$: not established

In unadjusted analyses, CRP and IL-6 were associated with increased odds of foot pain and forefoot pain among men. CRP was also associated with increased odds of hindfoot pain among women. TNF-α was not associated with foot pain or any structural foot disorder outcome. No other significant associations between inflammatory markers and foot outcomes were observed. After adjustment, CRP and IL-6 remained significantly associated with foot pain in men, and IL-6 with forefoot pain in men. No associations remained statistically significant in women (Table 2).

**Table 2:** Odds ratios and 95% CI for the association between inflammatory markers (logarithmic scale) and foot outcomes among men and women in the Framingham Foot Study

	Unadjusted				Adjusted^a^			
	Men		Women		Men		Women	
	OR	*P*-value	OR	*P*-value	OR	*P*-value	OR	*P*-value
**Foot pain**								
CRP	1.5 (1.1, 2.0)	< 0.01	1.3 (1.0, 1.5)	0.02	1.5 (1.1, 2.0)	0.01	1.1 (0.8, 1.3)	0.67
TNF-α	1.3 (0.6, 2.7)	0.46	1.0 (0.6, 1.6)	0.89	1.5 (0.7, 3.1)	0.31	1.0 (0.6, 1.6)	0.99
IL-6	1.8 (1.2, 2.6)	< 0.01	1.0 (0.7, 1.3)	0.89	1.8 (1.2, 2.6)	< 0.01	0.8 (0.6, 1.1)	0.22
**Forefoot pain**								
CRP	1.9 (1.1, 3.2)	0.03	1.1 (0.9, 1.4)	0.41	1.7 (0.9, 3.1)	0.12	1.0 (0.7, 1.3)	0.75
TNF-α	1.3 (0.3, 6.0)	0.76	0.9 (0.5, 1.8)	0.82	1.6 (0.3, 7.9)	0.55	1.0 (0.5, 1.9)	0.94
IL-6	2.4 (1.2, 4.7)	0.01	1.1 (0.8, 1.7)	0.53	2.9 (1.4, 6.1)	0.01	1.0 (0.7, 1.6)	0.87
**Hindfoot pain**								
CRP	1.2 (0.8, 1.7)	0.48	1.8 (1.2, 2.6)	0.003	1.2 (0.8, 1.9)	0.41	1.4 (0.9, 2.2)	0.13
TNF-α	1.1 (0.4, 3.3)	0.88	0.4 (0.1, 1.0)	0.06	1.3 (0.4, 4.0)	0.65	0.4 (0.1, 1.1)	0.07
IL-6	1.5 (0.9, 2.6)	0.14	1.4 (0.8, 2.3)	0.25	1.7 (1.0, 2.9)	0.07	1.1 (0.6, 1.9)	0.85
**Hallux valgus**								
CRP	0.8 (0.7, 1.0)	0.09	0.9 (0.8, 1.0)	0.16	0.8 (0.6, 1.0)	0.09	0.9 (0.7, 1.1)	0.22
TNF-α	1.1 (0.6, 2.0)	0.83	0.7 (0.5, 1.1)	0.14	1.0 (0.5, 1.9)	0.94	0.6 (0.4, 1.0)	0.06
IL-6	1.1 (0.8, 1.6)	0.45	0.9 (0.7, 1.2)	0.40	1.1 (0.8, 1.6)	0.49	0.9 (0.7, 1.2)	0.33
**Hallux rigidus**								
CRP	1.2 (0.7, 1.9)	0.57	0.9 (0.5, 1.6)	0.77	0.9 (0.5, 1.6)	0.76	1.2 (0.7, 2.3)	0.53
TNF-α	2.1 (0.7, 6.2)	0.19	1.2 (0.3, 5.3)	0.78	2.2 (0.7, 7.0)	0.20	1.3 (0.3, 5.8)	0.71
IL-6	1.0 (0.5, 2.0)	0.99	0.8 (0.3, 2.1)	0.59	0.7 (0.3, 1.7)	0.41	1.0 (0.4, 2.6)	0.94
**Toe deformity**								
CRP	1.0 (0.8, 1.3)	0.88	1.1 (0.9, 1.3)	0.24	0.9 (0.7, 1.2)	0.46	1.1 (0.9, 1.3)	0.50
TNF-α	1.2 (0.7, 2.2)	0.49	1.2 (0.7, 1.8)	0.49	1.0 (0.5, 1.8)	0.94	1.0 (0.6, 1.6)	0.97
IL-6	1.1 (0.8, 1.5)	0.47	1.1 (0.8, 1.5)	0.50	1.0 (0.7, 1.4)	0.80	0.9 (0.7, 1.3)	0.65

*Abbreviations: BMI* Body mass index, *CRP* C-reactive protein, *IL-6* Interleukin-6, *TNF-α* Tumor necrosis factor alpha

^a^ Odds ratios are adjusted for age, body mass index (BMI), smoking status, and physical activity index

Statistically significant associations (*p* < 0.05) in bold face. Models for TNF-α include a subset of 299 men and 348 women

No interactions between BMI and inflammatory markers were statistically significant (*p* > 0.15 for all interactions, data not shown).

## Discussion

In this cross-sectional study, inflammatory markers were associated with foot pain in men. After adjustment for confounding, CRP and IL-6 were associated with foot pain in men, and IL-6 was additionally associated with forefoot pain in men. No inflammatory markers were associated with structural foot disorders, and no inflammatory markers were associated with foot pain in women after adjustment.

While the biological basis for these findings remains an important area of investigation, prior research on musculoskeletal pain has noted differences in pain prevalence, pain severity, and pain risk factors by sex [5, 20, 21]. Women may report more musculoskeletal pain [21] and have a higher prevalence of foot pain [5]. Proposed mechanisms include differing physiologies of pain receptors and response by sex, as well as sociocultural factors impacting how pain is perceived and reported [21]. Additional work has suggested that, given the relationship between fat mass and foot pain, differences in body composition and fat mass distribution between men and women may also contribute [16].

The present work examined whether inflammatory markers may be associated with foot pain and structural foot disorders in a cross-sectional setting. The associations assessed in this study may help elucidate mechanisms of the established relationship between obesity and foot pain [4–6]. The physiological mechanisms underlying the association between obesity, inflammatory markers and pain are complex and not fully understood. CRP is a sensitive but non-specific marker of inflammation, and it has been demonstrated that increased circulating CRP is associated with increased pain sensitivity [22]. TNF-α, and IL-6, both pro-inflammatory cytokines, contribute to cartilage degradation in osteoarthritis and facilitate pain initiation and persistence. All three markers are elevated in obesity, which supports that our results warrant further exploration in relation to previously reported associations between fat mass and foot pain after adjustment for skeletal muscle mass [4]. No associations were observed between inflammatory markers and structural foot disorders, which suggests that the link between inflammation and foot pain is not mediated by alterations in foot morphology.

The etiology of foot pain has implications for interventions to improve mobility and maintain independence. When foot problems emerge due to mechanical load or structural foot disorders, footwear and orthoses may help [1]. However, these interventions may not be as effective in cases where inflammation may be the primary cause of foot pain. The relative benefits of mechanical versus pharmacological treatments in managing foot pain in the presence of obesity and inflammation warrants further investigation.

There are several limitations to this study. First, the cross-sectional nature of the study does not allow for confirmation of the temporal sequence between increases in inflammatory markers and the onset of foot pain. Second, this study controlled only for BMI, and not for body composition. Future work exploring the association between inflammatory markers and foot pain with respect to body composition is warranted. Additionally, foot and hindfoot pain may arise from multiple etiologies (e.g., fat pad insufficiency, plantar fasciitis, or shoe type). The relationship between inflammation and foot pain may differ based on such etiologies, with inflammation causing or mediating pain by different mechanisms and to different degrees. Further research with more detailed case definitions is necessary to explore these potential pathways. Due to low numbers, we were unable to control for several comorbidities (type II diabetes, depression, and inflammatory arthritis) that may be associated both with inflammation and pain. Additionally, inflammatory marker data was collected prior to the foot examination. While inflammatory markers have been shown to be stable over time, this analysis was unable to account for changes in inflammatory marker levels that may have occurred between the time of marker measurement and the FFS exam. Additionally, changes in weight or disease status may have led to trends in inflammation between the time of inflammatory marker measurement and foot assessment that may have impacted outcomes. Finally, the study sample used in this analysis is primarily composed of white individuals from the northeastern United States; replication of these findings in racially and geographically diverse samples is needed to assess the generalizability of these results. Strengths of the study include large numbers of community-dwelling men and women with comprehensive assessments of foot conditions and foot pain. These results point towards areas of future investigations in the relationship between inflammation in foot pain, particularly in older men.

## Conclusion

We found evidence that CRP and IL-6 are associated with foot pain in men, and that IL-6 is additionally associated with hindfoot pain in men. These findings support inflammation as having a potential role in the relationship between obesity and foot pain. While additional work confirming these associations in a longitudinal setting is necessary for determining the precise mechanism involved, these results indicate that further study of inflammation may be important for preventing and mitigating foot pain and its impacts on independence and quality of life.

## Supplementary Information

**Additional file 1: Supplementary Figure 1. **Participant inclusion flowchart. Eligible study participants had available inflammatory marker data form a Framingham Offspring Study visit and completed a Framingham Foot Study exam between 2002 – 2008. Participants were included if they had available data on C-reactive protein, interluekin-6, body mass index, sex, age, and physical activity index (PAI). Abbreviations used: TNF-α: tumor necrosis factor alpha.

## Data Availability

Data used in these analyses may be obtained upon reasonable request from the National Heart, Lung, and Blood Institute’s Framingham Heart Study at https://www.framinghamheartstudy.org/fhs-for-researchers/.
