# Supplementary material for: Foot pain and inflammatory markers: a cross sectional study in older adults
Source: J Foot Ankle Res. 2022 Aug 8;15:57. doi: 10.1186/s13047-022-00565-0 (PMC9358892; doi:10.1186/s13047-022-00565-0)
Supplement: Supplementary file 1 — Additional file 1: Supplementary Figure 1. Participant inclusion flowchart. Eligible study participants had available inflammatory marker data form a Framingham Offspring Study visit and completed a Framingham Foot Study exam between 2002 – 2008. Participants were included if they had available data on C-reactive protein, interluekin-6, body mass index, sex, age, and physical activity index (PAI). Abbreviations used: TNF-α: tumor necrosis factor alpha. [file 13047_2022_565_MOESM1_ESM.docx]

Inflammatory markers are related to foot pain in men but not structural foot disorders: the Framingham Foot Study

**Supplementary materials**


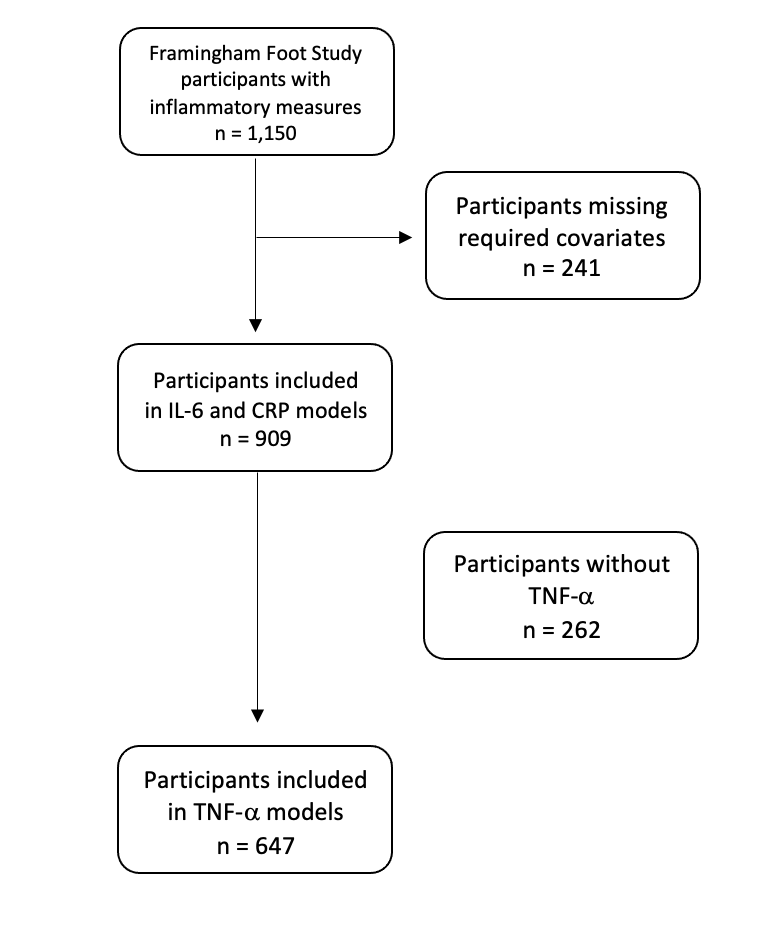


**Supplementary Figure 1.** Participant inclusion flowchart. Eligible study participants had available inflammatory marker data form a Framingham Offspring Study visit and completed a Framingham Foot Study exam between 2002 – 2008. Participants were included if they had available data on C-reactive protein, interluekin-6, body mass index, sex, age, and physical activity index (PAI). Abbreviations used: TNF-α: tumor necrosis factor alpha.
